# New Evidence of Semi-Mangrove Plant *Barringtonia racemosa* in Soil Clean-Up: Tolerance and Absorption of Lead and Cadmium

**DOI:** 10.3390/ijerph191912947

**Published:** 2022-10-10

**Authors:** Fang Liang, Ju Hu, Bing Liu, Lin Li, Xiuling Yang, Caihong Bai, Xiaohui Tan

**Affiliations:** 1 College of Biology and Pharmacy, Yulin Normal University, Yulin 537000, China; 2 Key Laboratory for Conservation and Utilization of Subtropical Bio-Resources, Education Department of Guangxi Zhuang Autonomous Region, Yulin Normal University, Yulin 537000, China; 3 Forestry of College, Guangxi University, Nanning 530001, China; 4 Guangxi Subtropical Crops Research Institute, Guangxi Academy of Agricultural Sciences, Nanning 530001, China

**Keywords:** *Barringtonia racemosa*, cadmium and lead stress, tolerance, absorption

## Abstract

Mangrove plants play an important role in the remediation of heavy-metal-contaminated estuarine and coastal areas; *Barringtonia racemosa* is a typical semi-mangrove plant. However, the effect of heavy metal stress on this plant has not been explored. In this study, tolerance characteristics and the accumulation profile of cadmium (Cd) and lead (Pb) in *B. racemosa* were evaluated. The results indicated that *B. racemosa* exhibited a high tolerance in single Cd/Pb and Cd + Pb stress, with a significant increase in biomass yield in all treatment groups, a significant increase in plant height, leaf area, chlorophyll and carotenoid content in most treatment groups and without significant reduction of SOD, POD, MDA, proline content, Chl a, Chl b, Chl a + b, Car, ratio of Chl a:b and ratio of Car:Chl (a + b). Cd and Pb mainly accumulated in the root (≥93.43%) and the content of Cd and Pb in *B. racemosa* was root > stem > leaf. Pb showed antagonistic effects on the Cd accumulation in the roots and Cd showed antagonistic or synergistic effects on the Pb accumulation in the roots, which depended on the concentration of Cd and Pb. There was a significant synergistic effect of Cd and Pb enrichment under a low Cd and Pb concentration treatment. Thus, phytoremediation could potentially use *B. racemosa* for Cd and Pb.

## 1. Introduction

Mangrove forests—woody plant communities that grow in the intertidal zone of tropical and subtropical coastlines [1]—provide a natural barrier to the coast [2]. Mangrove forests have an important ecological service value in the estuarine and coastal zones, where they contribute to plant community composition, play a role as a windbreak and in wave elimination, and contribute to phytoremediation and the removal of heavy metals [1,3,4,5].

Heavy metal pollution in mangrove forests has been widely reported. In the coastal zone of Olkhon Island, cadmium (Cd) and lead (Pb) could rise to dangerous levels [6]. Araujo et al., found the metal concentrations in soil fractions and mangrove organisms in the Botafogo estuary in Brazil exceeded by up to 2.6-fold the geochemical background, and 64% of the soils were contaminated with Pb [7]. Chen et al., found that Cd in sediments of mangrove forests could cause serious ecological risks [8]. El Ashmawy et al., indicated the contamination of sediment by Pb, Cu and Mn on the Egyptian Red Sea coast [9]. The presence of heavy metals in mangrove wetlands can affect soil microbial communities, soil organic matter decomposition and greenhouse gas emissions [10] and can reduce biodiversity [11] and damage human health [12]. At present, there are 20 families, 27 genera and 70 species of mangrove plants that have been reported around the world [13]. However, only a handful of species, namely *Acanthus ilicifolius* [14,15], *Rhizophora mucronata* [16], *Kandelia obovate* [14,17,18], *Bruguiera gymnorrhiza* [17,19,20], *R.stylosa*, *Aegiceras corniculatum* [19,20,21], *Avicennia marina* [9,14,20,22], *Excoecaria agallocha*, *Sonneratia apetala*, *S. caseolaris* [23] and *Heritiera littorlis* [9,24] have been found to tolerate Ca and enriched heavy metals such as Cd and Pb in polluted coastal zones. Therefore, the identification of mangrove plants suited for heavy-metal-contaminated areas, such as estuaries and coastal zones, is imperative for effective phytoremediation, the improvement of marine and terrestrial ecosystems and the protection of mangrove plant resources in the mangrove wetland.

*Barringtonia racemosa* is a typical semi-mangrove plant belonging to the Lecythideceae family that is commonly found throughout Eastern Africa, Polynesia, Africa and Asia [4,25,26]. In China, *B. racemosa* is mainly found in Hainan Island, Leizhou Peninsula and Guangdong province. The plant can strongly tolerate flooding and salinity stress environments [27,28,29], which makes it a great prospect for the restoration of mangrove wetlands as well as a significant windbreaker in the coastal zone wind and sand fixation. Around the coastal areas of Guangxi province, the Leizhou Peninsula and the northwest coast of Hainan Island, relatively high concentrations of Cd (0.03 to 0.12 mg kg^−1^) and Pb (16.99 to 57.98 mg kg^−1^) have been reported [30]. On the riparian zone and the estuary of Nanliujiang River in Guangxi province, the potential ecological risk of riparian zone soil in the Nanliujiang River rank as Cd > Pb > Cu > Zn [31], and high concentrations of Cd (12.57 mg kg^−1^) and Pb (83.76 mg kg^−1^) were found. Therefore, it is urgent to know *B. racemosa’s* tolerance and accumulation characteristics to heavy metal (especially Cd and Pb) contamination. The aim of this study was to study the tolerance and absorption ability of *B. racemosa* to Cd and Pb under single and compound stress.

## 2. Materials and Methods

### 2.1. Materials

*B. racemosa* seeds were collected from the natural *B. racemosa* forest in Danzhou city, Hainan Province, China, in October 2019. The seeds were sown in a sand bed in a greenhouse (22°64′ N, 110°14′ E) in the southeast Guangxi Flower Breeding Base of Yulin Normal University. Upon reaching a height of about 10 cm, the seedlings were transplanted into experimental pots (30 cm diameter and 28 cm height) containing a 1.5 kg mixture of culture substrate; the culture substrate was a soil, coconut bran and nutrient soil mixture at a ratio of 2:1:1 (v:v:v), with 1 plant per pot. The soil was taken from the 20 cm surface soil on the shore of Lake Yulan of Yulin Normal University, air dried, handpicked to remove plant detritus and stones, then used for plant culture. The physicochemical properties of the soil were as follows: organic matter, 30.52 g kg^−1^; total N, 2.04 g kg^−1^; total P, 0.72 g kg^−1^; total K, 7.47 g kg^−1^; pH, 7.62; Cd, 0.149 mg kg^−1^; Pb, 3.64 mg kg^−1^. The seedlings were used for the experiment after half a year of normal growth.

### 2.2. Pot Experiment

A total of 13 treatments were set up as described in Table 1. The concentrations of Cd and Pb added were based on the pre-experimental results, while the soil environmental quality risk control was according to the standard for soil contamination of agricultural land (Beijing, China, GB 15618-2018) [32,33]. Cd and Pb solutions were prepared by dissolving Cd (NO_3_)_2_·4H_2_O and Pb (NO_3_)_2_ in deionized water, respectively, then applying 400 mL of different treatment solutions to each pot with seedlings. To prevent the leakage of Cd and Pb solutions from the pots, all pots were placed in plastic trays, with three pots per tray. There was a total of 15 *B. racemosa* seedlings per treatment (1 seedling per pot), with 3 replicates. The soil was air dried, ground and passed through a 0.149 mm (100 mesh) nylon sieve. Next, 50 g of the soil was analyzed for heavy metal content as previously described [34,35].

### 2.3. Measurements and Analysis

#### 2.3.1. Measurement of Growth Indices

After 80 days of exposure to Cd and Pb stress treatments, plant height and ground diameter were determined by the method described by Liang et al., [29]. *B. racemosa* leaves and root morphology were scanned on the Microtek ScanMaker i800 plus system (WSeen, Hangzhou, China) and leaf area and root surface area were calculated using a LA-S Leaf Area and Root Morphology Analysis software (WSeen, Hangzhou, China), respectively.

One seedling from each pot was divided into its root, stem and leaf parts. Fresh tissue was weighed, dried at 105 °C for 30 min and samples were dried at 70 °C to a constant weight (approximately 48 h). Next, dry weights (DW) of the samples were recorded and the total biomass as well as root:shoot ratios (R/S) were calculated using the following formulae [18]:

Biomass = DW_root_ + DW_stem_ + DW_leaf_ (1)

R/S = DW_root_/(DW_stem_ + DW_leaf_) (2)

where DW_root_, DW_stem_ and DW_leaf_ represent the dry weight of the roots, stems and leaves, respectively.

#### 2.3.2. Determination of Superoxide Dismutase and Peroxidase Activity and Quantification of Malondialdehyde and Proline Levels

Three mature leaves of each plant were collected after 80 days of exposure to Cd and Pb stress treatments. The leaf samples (0.5 g) were ground under liquid nitrogen and homogenized in 10 volumes of ice-cold 50 mM sodium phosphate buffer (pH 7.8). The contents were centrifuged at 15,000× *g* at 4 °C for 20 min and the resulting supernatants were collected and used for enzyme activities. Superoxide dismutase (SOD) activity was determined using the modified nitroblue tetrazolium (NBT) method, peroxidase (POD) activity was determined by the guaiac wood phenol method, while enzyme activity was measured as described by Saleem [36]. Quantification of malondialdehyde (MDA) content was determined using the thiobarbituric acid (TBA) reaction method, as described by Atabaki et al. [37], while proline content was measured by the acid ninhydrin method [38]. The optical density (OD) values of SOD, POD, MDA and proline were measured with a Varioskan Lux Multimode Microplate Reader (Thermo Fisher Scientific, Singapore) [29].

#### 2.3.3. Photosynthetic Pigments Determination

Chlorophyll a (Chl a), chlorophyll b (Chl b) and carotenoid (Car) were extracted from 0.5 g of fresh leaves using 80% (*v*/*v*) acetone and quantified as previously described [39]. Extraction procedures were done at room temperature, under dark conditions for 24 h. Then chlorophyll a, b and Car content in the supernatant were measured using the Varioskan Lux Multimode Microplate Reader (Thermo Fisher Scientific, Singapore) at 649, 665 and 470 nm wavelengths, respectively. Concentrations of chlorophyll a, b, chlorophyll (a + b) and carotenoids in the leaf were calculated using the following equations [40,41]:

Chl a (mg g^−1^ FW) = 11.63 × A_665_ − 2.39 × A_649_ (3)

Chl b (mg g^−1^ FW) = 20.11 × A_649_ − 5.18 × A_665_ (4)

Chl (a + b) (mg g^−1^ FW) = 6.45 × A_665_ + 17.72 × A_649_ (5)

Car (mg g^−1^ FW) = (1000 × A_470_ − 1.82 × Chla − 85.02 × Chlb)/198 (6)

where FW denotes the fresh weight, while A indicates optical density.

#### 2.3.4. Determination of Cd and Pb Concentration

Cd and Pb standard solutions were purchased from the National Analysis Center for Iron and Steel, China Iron & Steel Research Institute Group (Beijing, China), while Cd (NO_3_)_2_·4H_2_O and Pb (NO_3_)_2_ reagents (minimum purity of 99.99%) were acquired from Guangxi Yulin Tianping Laboratory Products Co. The concentrations of Cd and Pb were measured according to the current Chinese National Standard GB5009.15-2014 and GB/T 2760 (2014), respectively. Roots, stems, leaves and soil samples were digested using HNO_3_-HClO_4_ [42]_,_ followed by the determination of the Cd and Pb concentration using a direct-reading inductively coupled plasma optical emission spectrometer ((ICP-OES) Optima8000, PerkinElmer Inc., Waltham, MA, USA) [14,43].

The translocation factor (TF) and bioenrichment factor (BCF) were calculated as follows:

Translocation factor (TF) = Cs/Cr(7)

Bioenrichment factor (BCF) = Cp/Cso(8)

where Cs is the metal concentration in the aerial part (shoot) of the plant, Cr is the concentration in the roots, Cp is the metal concentration in the plant (shoot) and Cso is the total metal concentration in the soil [44,45,46].

#### 2.3.5. Statistical Analysis

Differences between different Cd, Pb and Cd + Pb treatments were assessed by a one-way analysis of variance (ANOVA), which was implemented in SPSS 19.0 software (SPSS, Inc., Chicago, IL, USA) with multiple comparisons performed using the Duncan’s test at a 5% significance level (*p* < 0.05). Regression analysis was performed to develop models for the absorption of Cd and Pb using OriginPro 2021 9.8.5.201 (OriginLab Inc., Northampton, MA, USA).

## 3. Results

### 3.1. Effects of Cd and Pb on Plant Growth

**Figure 1:**
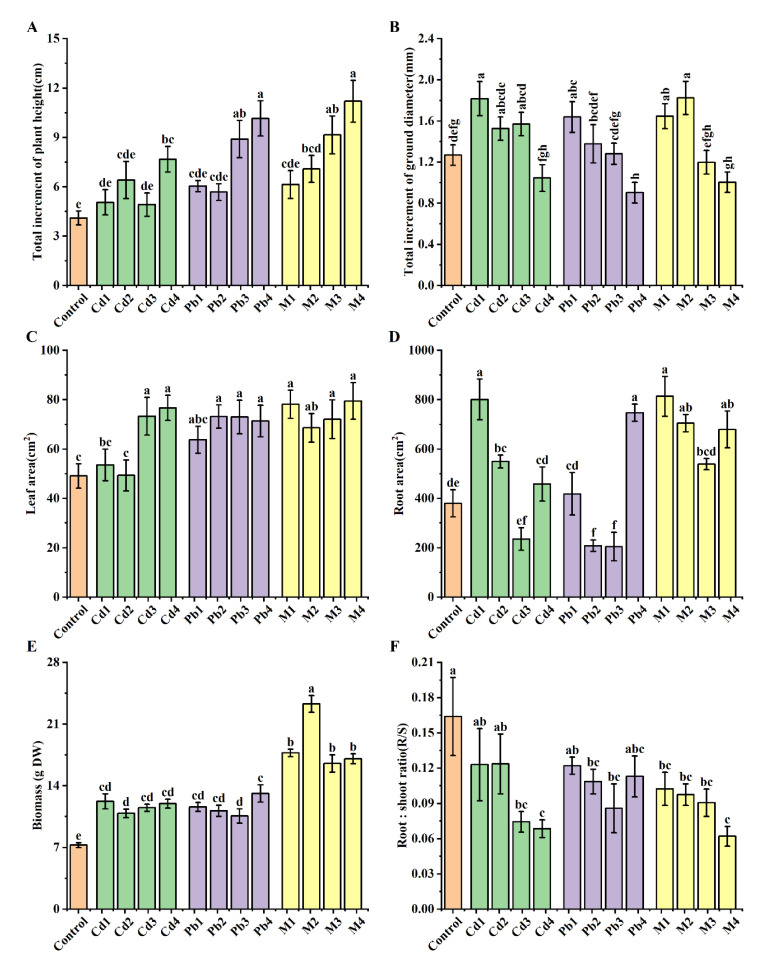
Effects of single Cd/Pb and Cd + Pb combined stress on growth indexes of *B. racemosa* seedlings. (**A**) Total increment of plant height (cm); (**B**) total increment of ground diameter (mm); (**C**) leaf area (cm^2^); (**D**) root area (cm^2^); (**E**) biomass (g DW); (**F**) root: shoot ratio (R/S). Means represent the average of three replicates (*n* = 3) ± SD, within each graph, mean values with common letters are not significantly different at *p ≤* 0.05 according to Duncan’s multiple range tests.

Cd stress increased plant height in *B. racemosa* (Figure 1A), while only the Cd4 treatment exhibited a significant increase (increased by 86.9%). Only the Cd1 treatment exhibited a significantly higher ground diameter (increased by 43.31%) relative to the controls (Figure 1B). Treatments with Cd3 and Cd4 resulted in a significant increase in leaf area, increasing by 49.27% and 56.20%, respectively, compared with the control (Figure 1C). Moreover, Cd1 and Cd2 resulted in a significantly higher root surface area, relative to the control, with the greatest effect observed in Cd1 (increased by 151.58%) (Figure 1D). With regard to biomass, *B. racemosa* plants under four Cd treatments increased significantly (Figure 1E). In addition, a significantly lower root:shoot ratio in plants under Cd3 and Cd4 treatments was found, relative to the control (Figure 1F).

For Pb stress, Pb3 and Pb4 treatments resulted in a significantly higher plant height (increased by 117.60% and 148.17%) relative to the control. Ground diameter significantly increased by 27.56% under the Pb1 treatment and decreased by 29.13% under the Pb4 treatment, compared with the control (Figure 1B). Moreover, plants exposed to three Pb treatments (Pb2, Pb3 and Pb4) had a significantly higher leaf area compared with the control (Figure 1C). Pb2 and Pb3 treatments resulted in a significantly lower root surface area (Figure 1D) and root:shoot ratio (Figure 1F), while plants treated with Pb4 exhibited a significant increase by 96.82% in root surface area (Figure 1D). Plants treated with four Pb concentrations exhibited a significant increase in biomass (Figure 1E)

For Cd + Pb treatment, the height of *B. racemosa* plants increased, with an increase in Cd + Pb concentration, compared with the control. The plant height under M2, M3 and M4 significantly increased; the highest value was observed in M4, evidenced by a 173.17% increment (Figure 1A). Exposure to M1 and M2 treatments resulted in a significant increase in ground diameter, 29.92% and 43.31% increments, respectively, relative to the control plants (Figure 1B). Plants treated with four Cd + Pb concentrations exhibited a significantly higher leaf area than the controls (Figure 1C). Moreover, plants treated with M1, M2 and M3 exhibited a significantly higher root surface area than their control (Figure 1D). *B. racemosa* plants exposed to four Cd + Pb treatments recorded a significantly higher biomass than the control; the highest value was observed in the M2 treatment (Figure 1E). Furthermore, exposure to four Cd + Pb treatments resulted in significantly lower root:shoot ratios compared with the control (Figure 1F).

### 3.2. Effects of Cd and Pb Stress on Physiological Characteristics

**Figure 2:**
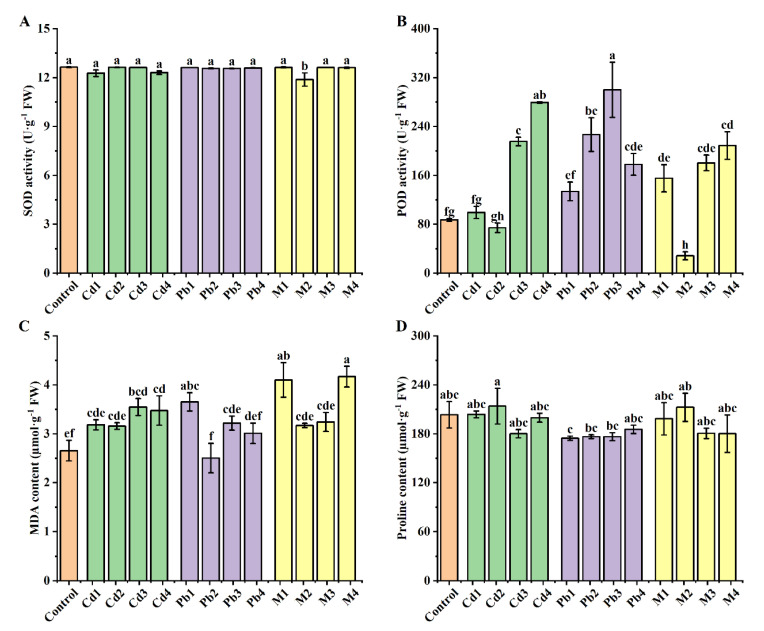
Effects of single Cd/Pb and Cd + Pb combined stress on leaf physiological indexes of *B. racemosa* seedlings. (**A**) Superoxide dismutase (SOD, U g^−1^ FW); (**B**) peroxidase activity (POD, U g^−1^ FW); (**C**) malondialdehyde content (MDA, µmol g^−1^ FW); (**D**) proline content (Pro, µmol g^−1^ FW). Means represent the average of three replicates (*n* = 3) ± SD, within each graph, mean values with common letters are not significantly different at *p ≤* 0.05 according to Duncan’s multiple range tests.

No significant differences were found in the superoxide dismutase (SOD) and proline concentrations across all Cd treatments and controls (Figure 2A,D). The plants treated with Cd3 and Cd4 had significantly higher peroxidase (POD) and malondialdehyde (MDA) than those in the control (Figure 2B,C), and POD under Cd4 stress was significantly higher than under the Cd3 treatment. There were no significant differences in MDA contents between plants treated with Cd3 and Cd4.

Similarly, there were no significant differences in SOD and proline concentrations across all Pb treatments and controls (Figure 2A,D). The plants treated with Pb2, Pb3 and Pb4 exhibited significantly higher POD levels than the controls, with the highest POD under the Pb3 treatment (Figure 2B). Exposure to Pb1 resulted in a significant increase in concentrations compared with the control (Figure 2C).

Significant lower SOD and POD concentrations were observed only after treatment with M2 compared with the control (Figure 2A,B). The plants treated with M1 and M4 had significantly higher MDA concentrations than the control (Figure 2C). No significant differences were observed in the proline concentration across the treatments (Figure 2D).

### 3.3. Effects of Cd and Pb Stress on Photosynthetic Pigments

**Figure 3:**
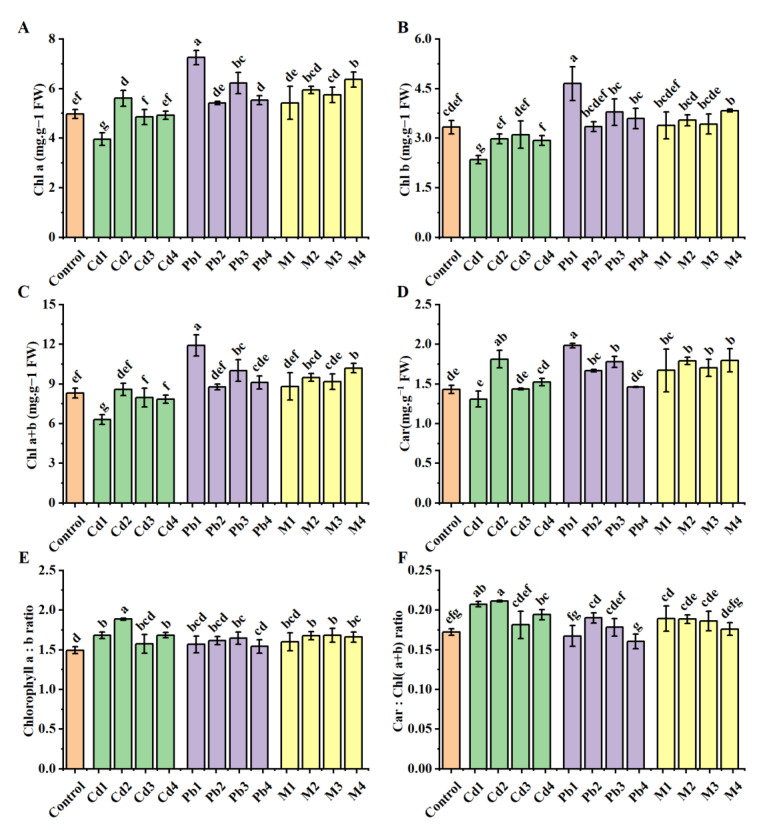
Photosynthetic pigments of *B. racemosa* under single Cd/Pb and Cd + Pb combined stress. (**A**) chlorophyll a (mg g^−1^ FW); (**B**) chlorophyll b (mg g^−1^ FW); (**C**) chlorophyll a + b (mg g^−1^ FW); (**D**) carotenoid (Car, mg g−1 FW); (**E**) chlorophyll a:b ratio; (**F**) Car:Chl (a + b) ratio. Means represent the average of three replicates (*n* = 3) ± SD, within each graph, mean values with common letters are not significantly different at *p ≤* 0.05 according to Duncan’s multiple range tests.

*B. racemosa* treated with Cd1 exhibited significantly lower chlorophyll (Chl) a, Chl b and Chl (a + b) concentrations than the control (Figure 3A–C). Cd1, Cd2 and Cd4 treatments significantly increased chlorophyll a:b ratio and Car:Chl (a + b) ratio (Figure 3E,F). *B. racemosa* treated with Pb1 exhibited significantly higher chlorophyll (Chl) a, Chl b, Chl (a + b) and Car concentrations than the control (Figure 3A–D). Pb3 and Pb4 treatments significantly increased Chl a (Figure 3A), Pb3 treatment increased Chl a + b (Figure 3C) and Pb2 and Pb3 treatments increased Car (Figure 3D). Pb3 and Pb2 treatments increased Chl a:b ratio and Car:Chl (a + b) ratio, respectively (Figure 3E,F). Under Cd + Pb treatments, compared with the control, M2, M3 and M4 significantly increased Chl a and chlorophyll a:b ratio (Figure 3A,E), M4 significantly increased Chl b (Figure 3B), M2 and M4 significantly increased Chl a + b (Figure 3C) and M1, M2, M3 and M4 significantly increased Carotenoid (Car) (Figure 3D). M1 significantly increased Car:Chl (a + b) ratio (Figure 3F).

### 3.4. Effects of Cd and Pb Stress on Enrichment Characteristics

#### 3.4.1. Concentration and Distribution of Cd and Pb in Plants

The Cd concentration and distribution in roots, stems and leaves of *B. racemosa* plants after 80 days of treatment are shown in Table 2. More than 93.43% of Cd in the plants accumulated in the roots under the Cd and the Cd + Pb treatments, indicating that Cd did not effectively translocate to stems and leaves. Cd accumulated less in the roots under the Cd + Pb treatment significantly than under the Cd treatment, exhibiting antagonistic effects. However, in the stems, Cd accumulated more under the M2 treatment significantly than under the Cd2 treatment, exhibiting synergistic effects. In the leaves, Cd accumulated more under the M4 treatment significantly than under the Cd4 treatment, exhibiting synergistic effects.

Similarly, Pb primarily accumulated in the roots (more than 95.20% of Pb in the plants) compared with the stems and leaves of *B. racemosa* seedlings under Pb and Cd + Pb treatments (Table 3). The results also indicated that Pb primarily accumulated in the roots and found it difficult to translocate to stems and leaves. Pb accumulation in the roots under M1 and M4 was higher than that under Pb1 and Pb4, respectively, which showed significant synergistic effects, while that under M2 and M3 in the roots was significantly lower relative to the plants exposed to Pb2 and Pb3, respectively, indicative of antagonistic effects. On the contrary, in the stems, Pb accumulation under M1 and M4 treatments were significantly lower than under Pb1 and Pb4 treatments, respectively, exhibiting antagonistic effects. Pb accumulation under M2 and M3 treatments were significantly higher than under Pb2 and Pb3 treatments, respectively, exhibiting synergistic effects. In the leaves, Pb accumulation under the M2 treatment was significantly lower than under the Pb2 treatment, exhibiting antagonistic effects. The Pb accumulation under the M1, M3 and M4 treatments were significantly higher than under the Pb1, Pb2 and Pb4 treatments, respectively, exhibiting synergistic effects.

Significantly high Cd and Pb enrichment coefficients were found under the M1 treatment (Table 2 and Table 3); the enrichment coefficient of Cd and Pb were 28.58 and 11.63, respectively. The translocation factor of Cd (0.35) and Pb (0.63) in the control was dramatically higher than under single Cd/Pb and Cd + Pb treatments (range 0.01–0.07 for Cd and 0.01–0.05 for Pb).

#### 3.4.2. Cd and Pb Absorption Models

**Figure 4:**
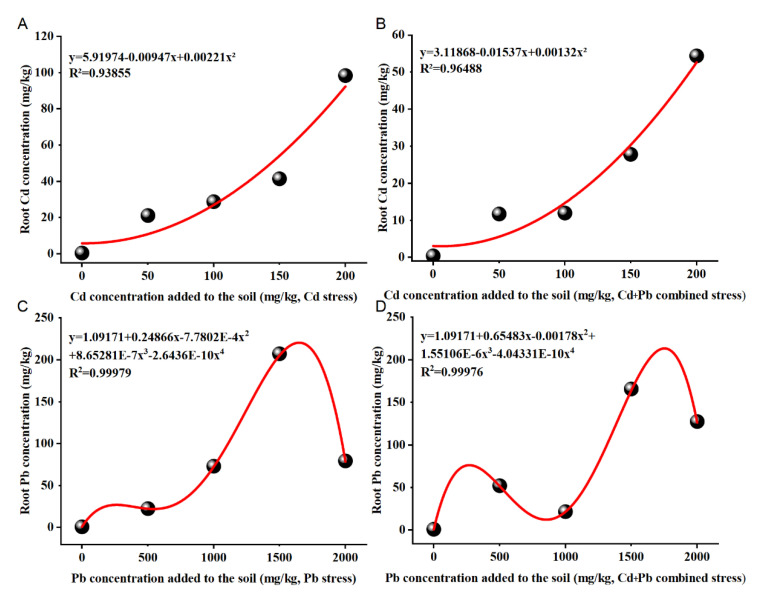
Models of absorption of Cd and Pb in *B. racemosa* under single Cd/Pb and Cd + Pb combined stress. (**A**) Cd absorption model under Cd stress; (**B**) Cd absorption model under Cd + Pb combined stress; (**C**) Pb absorption model under Pb stress; (**D**) Pb absorption model under Cd + Pb combined stress.

Results from the regression analyses revealed that the concentration of Cd and Pb in the soil under single Cd or Pb or Cd + Pb treatments were strongly correlated with (R^2^ ≥ 0.93) Cd or Pb in the roots (Figure 4A–D). The Cd concentration in the roots and the Cd concentration added in the soil fitted the quadratic equation under the Cd and Cd + Pb treatments. The Pb concentration in the roots and the Pb concentration added in the soil fitted the quartic equation in the Pb and Cd + Pb treatments.

## 4. Discussion

Tolerance, absorption and migration of mangrove plants to heavy metals is important for using mangrove plants for phytoremediation [15]. In the long evolutionary process, plants have formed their own internal anti-stress mechanism to reduce or eliminate the damage caused by various stresses. Mangrove plants *Kandelia obovate* and *B. gymnorrhiza* are tolerant to multiple heavy metals, such as Cd^2+^, Pb^2+^ and Hg^2+^ [19,47]. Mangrove plant *A. marina* is used in the phytoremediation process, owing to its ability in the deposition of heavy metals and the transport to upper plant parts, hence preventing pollution of the nearby areas with heavy metals [9].

Biomass, plant height and leaf area are important indicators of plant tolerance [48]. In the present study, typical semi-mangrove plant *B. racemosa* seedlings exposed to single Cd/Pb and Cd + Pb combined stress for 80 days did not exhibit heavy metal poisoning in either shoots or roots. Biomass was significantly increased under all single Cd/Pb and Cd + Pb stresses. Plant height and leaf area were significantly increased under most single Cd/Pb and Cd + Pb stresses. Ground diameter and root surface area did not significantly reduce under most single Cd/Pb and Cd + Pb stresses (Figure 1). These results, especially for biomass, indicated that *B. racemosa* exhibited strong stress tolerance against Cd and Pb stresses; probably, the increase of leaf area was for the purpose of absorbing more light energy and obtaining more organic matter to cope with the stress.

The antioxidant system in plants is a well-known defensive mechanism against reactive oxygen species and POD activity in roots and leaves maybe serve as a biomarker of heavy metal stress in *K. candel* [19,47]. Ozfidan-Konakci et al., and Doncheva et al., reported that the POD activity of *Kandelia obovate,* sunflower cultivated *H. annuus* cv. 1114 and interspecific line *H. annuus* × *H. argophyllus* increased to alleviate the toxicity of Cd and Pb [49,50]. MDA is an important product of cell membrane lipid peroxidation and it is an effective indicator of the degree of cell membrane damage [51]. In this study, compared with the control, the activity of POD increased significantly under high Cd and Pb concentration treatments, MDA significantly increased under Cd3, Cd4, Pb1, M1 and M4 (Figure 2) and *B. racemosa* showed strong resistance to high Cd and Pb stress. Significant lower POD concentrations were observed only under treatment with M2. SOD only under M2 treatment was significantly lower than the control group, which indicated that there were other ways or other cell osmotic substances to alleviate the toxicity of Cd and Pb.

Heavy metal stress affects the water balance in plants and induces a significant increase in the level of proline, which is involved in the osmotic regulation in cells [52]. However, there was no significant difference in the proline content in all treatment groups compared with the controls in the present study, probably because *B. racemosa* alleviates the damage of cell membrane permeability mainly through the enzyme system rather than by increasing the proline content. This also could be the strategy for the underlying response to heavy metals Cd and Pb in the *B. racemosa* species.

Photosynthetic pigments are essential for capturing light energy in plants, thus playing a key role in plant photosynthesis [53]. The chlorophyll content in photosynthetic pigments can directly reflect the growth status and the photosynthetic capacity of plants. Carotenoids are photosynthetic pigments that play important antioxidant roles; carotenoids can absorb residual energy, quench reactive oxygen species and prevent membrane lipid peroxidation [40]. Car/Chl (a  +  b) play key roles in protecting PSI and PSII photosystems [54]. Increasing the chlorophyll content possibly could reduce oxidative damage of the cell membrane by regulating the activities of antioxidant enzymes in wheat seedlings under Cd stress [53]. Previous findings indicated that heavy metal Pb and Cd stress reduces the chlorophyll content in the leaves of the mulberry (*Morus alba* L.) [55]. In the current study, the contents of Chl a, Chl b and Chl (a + b) significantly reduced only under Cd1 stress; Car, Chl a/b and Car/Chl (a + b) did not reduce under any Cd, Pb or Cd + Pb stress (Figure 3). These results could contribute to the tolerance to Cd and Pb contamination; Cd, Pb or Cd + Pb contamination could not reduce the antioxidant level or photosynthetic capacity of *B. racemosa*.

In the present study, more than 93.43% of Cd and Pb accumulated in the root system of the plants under Cd, Pb or Cd + Pb stress, and the content of Cd and Pb in *B. racemosa* was root > stem > leaf. Therefore, Cd and Pb primarily accumulated in the roots. The translocation factors (TF) of Cd and Pb were less than 1 under Cd, Pb and Cd + Pb stress (Table 2 and Table 3), the transport capacity of Cd and Pb was weak and Cd and Pb were difficult to transfer from the root to the aerial parts. However, the Cd and Pb enrichment ability of the root system of *B. racemosa* is stronger, which could be one of the reasons that *B. racemosa* was tolerant to Cd and Pb.

The coexistence of heavy metals is an important factor that affects the adsorption and transport of heavy metals in plants [9,46,56]. Liu et al. [57] reported that Cd inhibited the accumulation of Pb in *Impatiens balsamina*, whereas Pb promoted the accumulation of Cd. Wang et al., found that the added Pb may enhance the efficiency of Cd phytoextraction in wheat and maize [58]. Lou et al., found that the presence of a low Cd level impacted positively towards tall fescue growth under Pb stress, while a high level of Cd impacted negatively [59]. In this study, Pb inhibited Cd accumulation in the root (Table 2). However, Pb accumulation in the root showed significantly synergistic effects under M1 and M4, and antagonistic effects under M2 and M3 (Table 3). Therefore, the effect of Cd on the Pb accumulation depended on the concentration of Cd and Pb, probably because the effect of Cd on the Pb accumulation performed differently under different levels of Cd + Pb stress, and different Cd concentrations could induce different mechanisms of Pb accumulation, which is worth further investigation. Under each single Cd/Pb or Cd + Pb treatments, there were differences of the effect of Pb on the Cd accumulation, and the effect of Cd on the Pb accumulation were found between the roots, stems and leaves, which indicated that the interactions of Cd and Pb could be opposite in different organs. In addition, the highest enrichment coefficient of Cd and Pb were 28.58 and 11.63 under the M1 treatment, respectively (Table 2 and Table 3), which was about 10–20 times higher than that under other treatments. This result indicated that the synergistic effect of Cd and Pb enrichment under a low Cd and Pb concentration treatment (M1) was significantly stronger than under other treatments. Therefore, the phytoremediation effect of *B. racemosa* will be better in a low concentration of Cd and Pb polluted areas.

In this study, the absorption model of Cd and Pb in *B. racemosa* was simulated to analyze their absorption rules. The Cd concentration in the roots and the Cd concentration added in the soil of *B. racemosa* fitted the quadratic equation in Cd and Cd + Pb treatments (Figure 4A,B); roots will accumulate more Cd under higher Cd and Cd + Pb stress. The Pb concentration in the roots and the Cd concentration added in the soil fitted the quartic equation in Pb and Cd + Pb treatments (Figure 4C,D), though the concentration of Pb fluctuated with Pb concentration increasing in the soil and the Pb concentration would possibly increase in the roots. This indicated that *B. racemosa* potentially has the ability to hyperaccumulate Cd and Pb in the roots.

## 5. Conclusions

*B. racemosa* showed a high tolerance of single Cd/Pb and Cd + Pb stress, with a significant increase in biomass yield in all treatment groups, with significant increases in plant height, leaf area, chlorophyll and carotenoid content in most treatment groups, and without significant reduction of SOD, POD, MDA, proline content, Chl a, Chl b, Chl a + b, Car, ratio of Chl a:b or ratio of Car:Chl (a + b).

The *B. racemosa* plant accumulates high amounts of Cd and Pb in the roots and the root system plays an important role in ameliorating Cd and Pb toxicity. There were antagonistic effects of Pb on Cd accumulation and the effect of Cd on Pb accumulation depended on the concentration of Cd and Pb. The synergistic effect of Cd and Pb enrichment under a low Cd and Pb concentration treatment was significant. *B. racemosa* potentially has a significant ability to hyperaccumulate Cd in the roots and it has a high potential for the phytoremediation of Cd- and Pb-polluted soil.

## Figures and Tables

**Table 1:** Treated concentrations of Cd and Pb.

Treatments	Cd (mg kg^−1^)	Treatments	Pb (mg kg^−1^)	Treatments	Cd + Pb (mg kg^−1^)
Control	0	-	0	-	0 + 0
Cd1	50	Pb1	500	M1	50 + 500
Cd2	100	Pb2	1000	M2	100 + 1000
Cd3	150	Pb3	1500	M3	150 + 1500
Cd4	200	Pb4	2000	M4	200 + 2000

**Table 2:** Concentration and distribution of Cd in roots, stems and leaves of *B. racemosa* under single Cd/Pb and Cd + Pb stress.

Treatment	Cd Concentration Mean ±SD (mg kg^−1^ DW) (Distribution (%))				Cd Enrichment Coefficient	Cd Translocation Factors
	Root	Stem	Leaf	Total		
CK	0.50 ± 0.01 (74.57) ^g^	0.00 ± 0.00 (0.00) ^g^	0.17 ± 0.00 (25.43) ^c^	0.66 ± 0.01 ^g^	2.54 ± 0.00 ^b^	0.35 ± 0.02 ^a^
Cd1	21.21 ± 0.53 (93.43) ^e^	1.37 ± 0.31 (6.06) ^a^	0.12 ± 0.00 (0.51) ^d^	22.70 ± 0.25 ^e^	2.51 ± 0.03 ^b^	0.07 ± 0.02 ^b^
Cd2	28.93 ± 2.81 (98.22) ^d^	0.37 ± 0.01 (1.27) ^ef^	0.15 ± 0.01 (0.51) ^c^	29.46 ± 2.82 ^d^	1.66 ± 0.16 ^c^	0.02 ± 0.00 ^ef^
Cd3	41.59 ± 0.41 (97.51) ^c^	0.81 ± 0.00 (1.90) ^b^	0.25 ± 0.01 (0.59) ^b^	42.66 ± 0.40 ^c^	1.41 ± 0.01 ^c^	0.03 ± 0.00 ^def^
Cd4	98.56 ± 1.10 (98.66) ^a^	1.26 ± 0.00 (1.26) ^a^	0.08 ± 0.01 (0.08) ^e^	99.90 ± 1.10 ^a^	1.80 ± 0.02 ^c^	0.01 ± 0.00 ^f^
M1	11.79 ± 0.09 (96.65) ^f^	0.31 ± 0.02 (2.52) ^f^	0.10 ± 0.03 (0.83) ^de^	12.20 ± 0.06 ^f^	28.58 ± 0.95 ^a^	0.03 ± 0.00 ^d^
M2	12.04 ± 0.034 (95.23) ^f^	0.50 ± 0.00 (3.98) ^de^	0.10 ± 0.01 (0.79) ^de^	12.64 ± 0.05 ^f^	1.21 ± 0.01 ^c^	0.05 ± 0.00 ^c^
M3	27.88 ± 0.07 (97.14) ^d^	0.70 ± 0.00 (2.44) ^bc^	0.12 ± 0.02 (0.43) ^d^	28.71 ± 0.07 ^d^	1.32 ± 0.00 ^c^	0.03 ± 0.00 ^de^
M4	54.41 ± 0.02 (97.61) ^b^	0.62 ± 0.04 (1.11) ^cd^	0.72 ± 0.00 (1.29) ^a^	55.74 ± 0.02 ^b^	1.76 ± 0.01 ^c^	0.02 ± 0.00 ^def^

Different letters indicate significance of *p* < 0.05. Letters shared in common indicate no significant difference.

**Table 3:** Concentration and distribution of Pb in roots, stems and leaves of *B. racemosa* under single Cd/Pb and Cd + Pb stress.

Treatment	Pb Concentration (mg/kg) (Distribution (%))				Pb Enrichment Coefficient	Pb Translocation Factors
	Root	Stem	Leaf	Total		
CK	1.10 ± 0.32 (61.44) ^g^	0.40 ± 0.17 (21.80) ^b^^c^	0.30 ± 0.05 (16.76) ^de^	1.80 ± 0.52 ^h^	0.47 ± 0.14 ^c^	0.63 ± 0.08 ^a^
Pb1	22.78 ± 0.35 (95.20) ^f^	0.95 ± 0.09 (3.95) ^a^	0.20 ± 0.07 (0.85) ^e^	23.93 ± 0.31 ^g^	0.53 ± 0.01 ^c^	0.05 ± 0.01 ^b^
Pb2	73.38 ± 0.39 (98.83) ^d^	0.39 ± 0.11 (0.52) ^bc^	0.48 ± 0.10 (0.18) ^bc^	74.25 ± 0.41 ^e^	0.69 ± 0.01 ^c^	0.01 ± 0.00 ^b^
Pb3	207.61 ± 11.21 (99.66) ^a^	0.31 ± 0.09 (0.15) ^c^	0.38 ± 0.07 (0.18) ^bcde^	208.31 ± 11.15 ^a^	1.51 ± 0.08 ^b^	0.00 ± 0.00 ^b^
Pb4	79.61 ± 2.84 (98.15) ^d^	0.98 ± 0.04 (1.20) ^a^	0.53 ± 0.18 (0.65) ^b^	81.11 ± 2.95 ^d^	0.40 ± 0.01 ^c^	0.02 ± 0.00 ^b^
M1	52.42 ± 0.47 (97.65) ^e^	0.94 ± 0.16 (1.74) ^a^	0.33 ± 0.07 (0.61) ^cde^	53.68 ± 0.57 ^f^	11.63 ± 0.58 ^a^	0.02± 0.00 ^b^
M2	21.97 ± 0.12 (95.99) ^f^	0.53 ± 0.04 (2.31) ^b^	0.39 ± 0.05 (1.70) ^bcd^	22.89 ± 0.14 ^g^	0.54 ± 0.00 ^c^	0.04± 0.00 ^b^
M3	165.87 ± 0.70 (99.44) ^b^	0.48 ± 0.12 (0.29) ^bc^	0.45 ± 0.21 (0.27) ^bc^	166.81 ± 0.88 ^b^	1.53 ± 0.01 ^b^	0.01 ± 0.00 ^b^
M4	127.61 ± 0.17 (99.06) ^c^	0.43 ± 0.13 (0.33) ^bc^	0.79 ± 0.03 (0.61) ^a^	128.82 ± 0.09 ^c^	1.24 ± 0.01 ^b^	0.01 ± 0.00 ^b^

Different letters indicate significance of *p* < 0.05. Letters shared in common indicate no significant difference.
